# Targeting the glycans: A paradigm for host‐targeted and COVID‐19 drug design

**DOI:** 10.1111/jcmm.16585

**Published:** 2021-05-24

**Authors:** Fatemeh Pourrajab

**Affiliations:** ^1^ Reproductive Immunology Research Center Shahid Sadoughi University of Medical Sciences Yazd Iran; ^2^ Nutrition and Food Security Research Center Shahid Sadoughi University of Medical Sciences Yazd Iran; ^3^ Biotechnology Research Center, International Campus Shahid Sadoughi University of Medical Sciences Yazd Iran

**Keywords:** coronavirus, glycan‐recognition agents, glycans, spike protein

## Abstract

There is always a need for new approaches for the control of virus burdens caused by seasonal outbreaks, the emergence of novel viruses with pandemic potential and the development of resistance to current antiviral drugs. The outbreak of the 2019 novel coronavirus‐disease COVID‐19 represented a pandemic threat and declared a public health emergency of international concern. Herein, the role of glycans for the development of new drugs or vaccines, as a host‐targeted approach, is discussed where may provide a front‐line prophylactic or threats to protect against the current and any future respiratory‐infecting virus and possibly against other respiratory pathogens. As a prototype, the role of glycans in the coronavirus infection, as well as, galectins (Gal) as the glycan‐recognition agents (GRAs) in drug design are here summarized. Galectins, in particular, Gal‐1 and Gal‐3 are ubiquitous and important to biological systems, whose interactions with viral glycans modulate host immunity and homeostatic balance.

## 1 INTRODUCTION

Virus infections are initiated by the attachment of viral particles to the protein or carbohydrate receptors on the host cell. It is an important determinant of viral host range and cross‐species infection and a primary target for antiviral intervention. Coronaviruses recognize a variety of host receptors (including protein or carbohydrate receptors) through the spike protein, infect many hosts and are health threats to humans and animals.^1^, ^2^, ^3^, ^4^

The 2019 novel SARS‐coronavirus 2 (SARS‐CoV‐2) which causing acute respiratory infection and responsible for the recent worldwide pandemic of coronavirus pneumonia (COVID‐19) is an enveloped virus that uses host glycosylation machinery to glycosylate its proteins such as spike glycoprotein (SGP) S. SARS‐CoV‐2 evolves unique N‐ and O‐linked glycosylation sites of SGP S that distinguish it from SARS and MERS coronaviruses. The glycan shielding underlines camouflage of SARS‐CoV‐2 from the host defence system and immune evasion. Like viral envelope proteins, the cellular receptor the virus has also glycan moieties which affect viral efficient binding to the cell surface and SGP‐triggered membrane fusion. A new type of ganglioside‐binding domain at the tip of the N‐terminal domain of the SGP S has been identified which was found fully conserved among clinical isolates of SARS‐Cov‐2 worldwide.^1^, ^3^, ^5^, ^6^

Out of a dozen or more potential drugs to treat COVID‐19 infection are already in clinical trials. One of them is the well‐known antimalarial drug, chloroquine (CLQ), and its alternative hydroxychloroquine (CLQ‐OH), which have attained notifications in clinics. In vitro and infected patients, both compounds displayed that can efficiently inhibit severe acute respiratory system Cov‐2 (SARS‐Cov‐2) infection. The mechanism that has been revealed until now is through increasing the low pH of intracellular endosomes (required for virus fusion), interfering with the host glycosylation process, as well as, masking sialoglycans at the cellular surface where blocking virus‐host receptor interaction and viral entry (needed for subsequent viral replication and syncytial formation).^7^, ^8^, ^9^ Identification of this new mechanism of action of CLQ and CLQ‐OH (interfering with the host glycosylation and masking sialoglycans) provides supports for the use of these repositioned drugs to cure patients infected with SARS‐Cov‐2.^3^, ^5^

There are reports for the prevention of influenza by intranasal targeting of host receptors using engineered proteins masking the cell surface glycans. Studies have designed multivalent biologics, engineered carbohydrate‐binding agents (CBAs) specific for sialic acid that use sialic acid as a receptor and mask the cell surface receptors. The biologics mask the cell surface receptors recognized by the influenza virus (IV) and could protect mice from a challenge with a lethal dose of 2009 pandemic H1N1 IV. However, there was sufficient virus replication to establish an immune response, potentially protecting the animal from future exposure to the virus.^10^, ^11^, ^12^

Additionally, there are reports for antiviral activity of iminosugars (eg in the case of HIV‐1), whose mechanism of action is through inhibiting ER glucosidases and glycosylation process of cell surface receptors. The ER‐glucosidase activity is required for glycosylation of cellular and viral receptors. The iminosugars potently inhibit the release of infectious virion particles and the number of infected cells, however, a barrier to their clinical application, is that they show off‐target effects whereby impeding host gut glucosidases leading to diarrhoea and abdominal pains. Additionally, viral spike glycoproteins (SGPs) are glycosylated by the host cell as able to hijack cellular glycosylation.^6^, ^13^

It is, therefore, possible that by the inhibition of the host cell glycosylation process or interfering with glycan interactions at the cellular surface, the SGPs are no longer able to bind to the host glycans.^3^, ^5^

Some mechanisms and features show the important role of glycans in viral infection, as well as, in the development of prophylactics and treatments especially for the development of new vaccines.^6^, ^12^, ^13^

Furthermore, the host‐targeted approach could provide a front‐line prophylactic that has the potential to protect against any current and future respiratory‐infecting virus and possibly against other respiratory pathogens.

## 2 RECEPTOR RECOGNITION MECHANISMS OF CORONAVIRUSES

The large spike (S) glycoprotein (GPS) (~200‐kDa) on the envelope of CoV virions binds to host‐specific receptors whereby mediate virus entry, tissue tropism, and host range; and can affect virus virulence. CoV GPS is class I viral fusion protein, like IV hemagglutinin (HA), human immunodeficiency virus‐1 (HIV‐1) envelope GP (Env), Ebola virus GP and paramyxovirus F GPs.^14^, ^15^

There are structural similarities between GPS of SARS‐CoV‐2, SARS‐CoV and MERS‐CoV that suggest the possibility for them to share receptors. In SARS‐CoV and SARS‐CoV‐2, the GPS exhibits similarity in structure and sequence of the RBD and the identity in residues critical for ACE2 binding, the majority of which are either highly conserved or shared similar side chain properties. SARS‐CoV and SARS‐CoV‐2 GPS exhibit an overall sequence identity of about 76%, with only 51% identity in NTD, 64% in RBD and 90% in the S2 fusion domain.^16^, ^17^, ^18^ In comparison, SARS‐CoV‐2 and MERS‐CoV share lower sequence identity in their spikes (~35%), RBDs, or RBM, and yet they recognize the same receptor dipeptidyl peptidase‐4 (DPP4). The GPS of the SARS‐CoV‐2 gene is longer than SARS‐CoVs and there are three short insertions in the N‐terminal domain, which may confer a sialic acid‐binding activity like as MERS‐CoV GPS.^1^, ^16^, ^17^, ^18^

CoV GPS is separated into two subunits, called S1 and S2, by cellular proteases (host or producing‐cell). It first binds to a receptor on the host cell surface through its S1 subunit and then fuses viral and host membranes through its S2 subunit.^14^, ^15^ The S1 subunit of GPS contains two distinctive domains, the N‐terminal domain (S1‐NTD) and the C‐terminal domain (S1‐CTD), both of which can function as receptor‐binding domains (RBDs). S1‐NTD is a carbohydrate‐binding domain, (CBD) responsible for recognizing and binding to sugars, while S1‐CTD binds protein receptors such as dipeptidyl peptidase‐4 (DPP4), angiotensin‐converting enzyme 2 (ACE2) and APN, and function as the functional‐binding domain (FBD).^1^, ^2^, ^3^, ^19^, ^20^ In the case of the SARS‐CoV‐2 and MERS‐CoV receptor, the human GP CD26 or DPP4 is a key immune‐regulatory factor for hijacking and virulence and is widely expressed on epithelial cells in the kidney, alveoli, small intestine, liver, and prostate, and activated leucocytes. These viruses might deregulate antiviral T cell responses due to the stimulation of T cell apoptosis.^1^, ^21^ In the case of the SARS‐CoV receptor, the membrane‐associated aminopeptidase ACE2 is highly expressed in the lung, vascular endothelia, renal and cardiovascular tissue, and epithelia of the small intestine and testes.^22^ ACE2 plays a crucial role in elderly people by regulating the RAS via opposing the actions of Ang II because it has a beneficial role in many diseases such as hypertension, diabetes and cardiovascular disease.^11^

Through S1‐CTD, SARS and MERS‐CoVs bind to ACE2 and DPP4, respectively, while the novel SARS‐CoV‐2 S1‐CTD seems able to utilize both receptors to infect host cells.^1^, ^2^, ^20^, ^23^ The S1 subunit is further divided into four β‐rich subdomains, designated as A, B, C and D, with subdomains A and B acting as RBDs in different coronaviruses.^17^, ^24^ In SARS‐CoV‐2 and MERS‐CoV, the S1B subdomain recognizes the host receptors ACE2 and DPP4, respectively, while viral initial binding and primary attachment is through the interaction between the S1A subdomain and host α2,3/6‐linked Sia. Herein, the S1B subdomain has a major influence on the virus‐host range and tissue tropism, and its receptor tissue localization varies between species.^1^, ^2^, ^5^, ^25^ Whereas, the S1A subdomain which participating in the early phase of viral attachment and infection shares conserved structural folds and sugar‐binding sites in different viral lectins.^4^, ^5^, ^24^, ^25^

Studies reveal that the S1‐NTD resembles the structural folds and sugar‐binding sites of human galectin‐3 (Gal‐3),^1^, ^20^, ^24^ whereas the S1‐CTD is aggressively evolving and exploits novel protein receptors to determine the viral specificity of receptor binding and host tropism.^14^, ^15^, ^26^ It appears to be a successful strategy for viruses to share a lectin structure as a sugar receptor and acquire RBDs with novel specificity or altered specificity for a protein receptor.^14^, ^16^, ^25^, ^27^

### 2.1 Glycans enrolled to participate in virus‐host interaction

Several articles have focused on glycosylation patterns that occurred in the context of several viral envelope GPs as they play an important role in viral infection and pathogenesis. These viral GPs are including the HIV‐1 GP Env, IV‐GP HA, CoV GPS, Ebola virus GP and sGP, GP complex of Lassa virus, and envelope (E) GP of dengue and Zika viruses. Viral GPs are mostly N‐linked glycans shielded by oligomannose‐type glycan clusters.^6^, ^13^, ^23^, ^28^ Viruses exploit host cell machinery to glycosylate their proteins during replication. Glycosylation depends on host cell machinery and plays an important role in the viral lifecycle, virulence and host immune responses to infection. Blocks of fourteen sugars (Glc3Man9GlcNAc2) constitute the N(Asn)‐linked glycosylation that is occurred co‐translationally on native polypeptides in the endoplasmic reticulum (ER). The blocks are then subjected to a series of modifications during their transport through the ER and the Golgi complex before reaching their final destinations inside or outside the cell. A group of transferases modifies the ends of glycans to galactose, fucose and sialic acids (Sia), to construct a huge assortment of different classes of glycans namely oligomannose, hybrid and complex‐type N‐glycans. The O(Ser/Thr/Tyr)‐linked glycosylation is primarily occurred in the Golgi apparatus and creates specific recognition sites or masking immunogenic epitopes on the proteins. O‐linked glycans usually pose N‐acetylgalactosamine (GalNAc) as the binding sugar but at the head can also involve other sugars, such as galactose, fucose, N‐acetylglucosamine (GlcNAc) and sialic acids (Sia).^6^, ^12^, ^13^ The CoV GPS, as well as, its host‐functional receptors (eg human ACE2 and DPP4) pose N‐linked glycans containing fucosylated core, galactose residues and various levels of sialylation (single or more, terminal Sia). The antiviral effect of iminosugars is through altering the N‐linked glycan structures of both viral and host GPs whose expressions on the cell surface are not affected but their interactions are interrupted.^6^, ^17^ Inhibition of ER glucosidases by iminosugars does not inhibit virus‐host GP expressions, but efficiently blocks virus replication and spreading through disrupting the glycan shield of the viral spikes required for virus‐host receptor interaction. Additionally, alteration of N‐linked glycans on the surface of host cells impairs their ability to support the transduction of an infectious virus such as CoVs and IV.^6^, ^7^, ^29^ In the case of virus infectivity, the glycosylation pattern can be compositionally different across different species and individuals, an important feature of viral interspecies/inter‐individual transmission potential where influences viral tropism.^6^, ^13^ Additionally, differential organization of viral glycosylation across viral GPs influences not only individual glycan compositions but also the immunological pressure across the viral protein surface that put them principal targets of the neutralizing humoral immune response.^17^, ^23^

**FIGURE 1:**
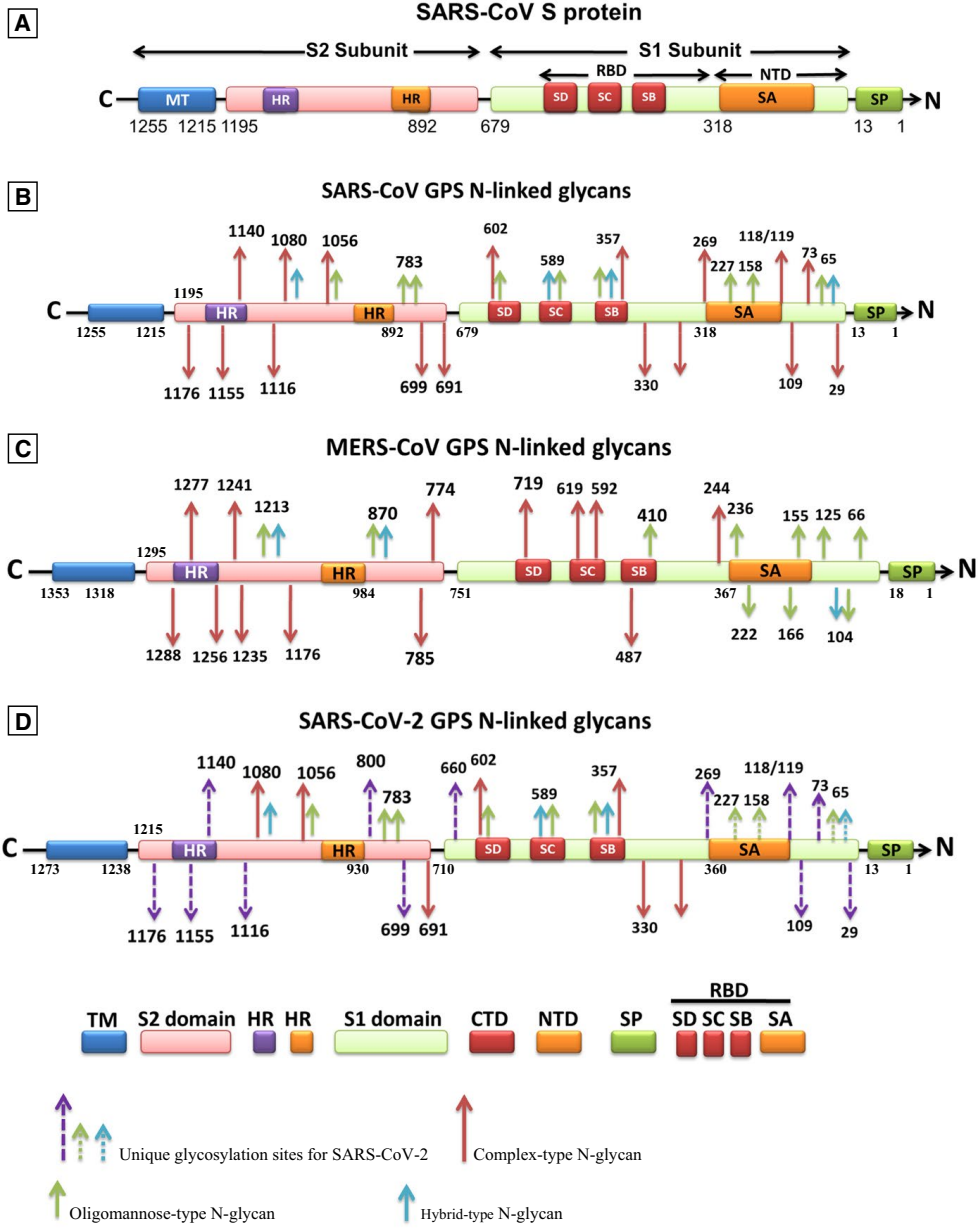
The predicted structures of the spike glycoprotein (SGP) S in coronaviruses (SARS‐CoV, MERS‐CoV, SARS‐CoV‐2 (A‐D)), and the glycosylation profiles of detected N‐linked glycans (B‐D). The SGP S structure can be divided into the S1 and S2 domains, and the structural domains in the spike protein are located in the order (from C to the N terminus) as: transmembrane (TM), heptad repeats (HRs) in the S2 domain, C‐terminal domain (CTD), and N‐terminal domain (NTD) in the S1 domain as well as the signal peptide (SP). The S1‐CTD is divided into three subdomains SD‐SB, while S1‐NTD contains subdomain SA. SD‐SA is accounted as receptor‐binding domain (RBD). (B‐D) Glycosylation sites for oligomannose, hybrid and complex‐type N‐glycans are coloured in green, blue and red, respectively. Unique glycosylation sites for SARS‐CoV‐2 are dashed in violet, green and blue, the dual recognition of gangliosides and angiotensin‐converting enzyme‐2 (ACE‐ 2) by SARS‐CoV‐2 spike (S) protein. The viral protein displays two distinct domains, the tips of which are available for distinct types of interactions (S1‐NTD). The receptor‐binding domain (S1‐CTD) binds to the ACE‐2 receptor, and the N‐terminal domain (NTD) binds to the ganglioside/sialoglycan‐rich domain of the plasma membrane. Lipid rafts, which are membrane domains enriched in gangliosides and cholesterol, provide a perfect attractive interface for adequately positioning the viral S protein at the first step of the infection process

In SARS‐CoV‐2, SARS and MERS‐CoV, the overall structure of GPS can resemble each other, but the density and compositions of glycan shielding are markedly different (Figure 1). Marked differences exist between the residues 40‐318 located at the subdomain S1A in S1‐NTD. Moreover, densely glycosylated spike proteins become topologically different in the position of the RBDs in their respective conformations and architectures of the S1 receptor‐binding subunits to bind to functional receptors and gain entry into host cells. A critical step in the crosstalk between the virus and the host cell is the binding of S‐glycans to the functional receptor on the surface of human cells.^1^, ^17^, ^23^ The N‐ and O‐linked glycosylation sites of SARS‐CoV‐2 GPS show some similarities to that of SARS‐CoV. However, there are unique N‐ and O‐linked glycosylation sites and compositions on GPS of these CoVs that distinguish them from each other. As well, different glycosylation and compositions do shielding and camouflage of CoVs from the host defence system.^1^, ^17^, ^23^, ^26^

#### 2.1.1 Viral glycans and engagement of immune receptors

Viral envelope proteins are glycosylated to varying degrees, but depending on their overall mass, surface area, and volume, the overall density of glycan shielding may differ significantly between different viruses. Additionally, the extensive glycosylation of SGPs masks viral immunogenic protein epitopes from the host humoral immune system by occluding them with host‐derived glycans. There is a high‐density glycan shield on the SARS, MERS and SARS‐CoV‐2 S that facilitates diverse structural and functional roles during the viral infection. There is an abundance of oligomannose‐type glycans at specific regions of high glycan density on MERS and SARS‐CoV‐2 S that is effective in viral evasion ability (Figure 1). A strong correlation has been observed between the ‘evasion strong’ type of virus and significantly elevated glycan shield densities and oligomannose abundance.^1^, ^23^ Herein, literature provides novel mechanisms by which the glycan shield of SARS‐CoV‐2 SGP S plays a strong role in immune evasion and virus attachment to the innate immune cells that enhanced viral infection.^1^, ^6^, ^23^

Engagement of innate immune receptors causes phagocytosis and engulfment of the virus by mature dendritic cells (DCs) and macrophages whereby facilitating and/or augmenting viral infection. Virus capture and storage by mature DCs and macrophages mediate of other immune cells, independently of peptidase receptors (phagocyte‐mediated trans‐infection).^30^, ^31^, ^32^

Viral GPs pose microbial signatures recognized by C‐type mannose‐binding lectins for example DC‐SIGN and L‐SIGN (dendritic cell‐specific ICAM3‐grabbing non‐integrin, liver/lymph node‐SIGN). Through binding to these receptors, viruses can capture phagocytes and transfect other target cells, as well as, exploiting innate and acquired immune functions. Viruses such as SARS and HIV‐1 use these receptors to capture DCs and through a trans‐infection mechanism transmitting to other target cells, especially promoting a vigorous infection of CD4+T cells.^6^, ^28^, ^30^ Evidence implies and highlights the most important role of sialyl lactose moiety on the glycoconjugates of viral membrane, as well as, the cellular Siglec‐1 (sialic acid‐binding immunoglobulin‐like lectins (Siglecs)) as critical determinants for viral capturing and infectivity of other cells.^3^, ^28^, ^31^ There are two determinants in HIV‐1 infection, sialyl‐lactose‐containing gangliosides in the viral membrane derived from the first host and the sialic acid‐binding immunoglobulin‐like lectin‐1 (Siglec‐1) located on the second host phagocytes or T cells. The cellular lectin Siglec‐1 is a critical determinant for phagocytes or T cells infection by HIV‐1. Paradoxically, the sialoglycan composition of the viral envelope and Siglec‐1 from host DCs contribute to viral pathogenesis and transmission, a prototype mechanism that can be attributed to other similar viruses.^6^, ^28^

The CoV spike proteins contain unique N‐ and O‐linked glycosylations that distinguish them from each other and underline shielding and camouflage of virus from the host defence system. Meanwhile, the hybrid and complex‐type of N‐linked glycans have been implicated to be in particular recognized by the innate immune cells.^1^, ^6^, ^23^

Herein, seven N(Asn)‐linked glycosylation sites (residues at positions 109, 118, 119, 158, 227, 589 and 699) have been identified in SARS‐CoV GPS, for binding to DC‐SIGN‐or L‐SIGN. These residues differ from those of the ACE2‐binding domain located at S1‐CTD, amino acids 318‐510.^3^, ^28^, ^31^ About 69N‐linked glycan sites have been identified in each monomer of GPS in HKU1, SARS and MERS‐CoV where 29, 23 and 23 of them were found glycosylated, respectively. There are also 66 sites (spanning S residues 27 to 1146) in SARS‐CoV‐2 GPS whose a total of 22N‐linked glycan sites were detected and 18 of them being in common with SARS GPS.^1^, ^23^ The N‐linked glycosylation sites contain relative quantities of oligomannose, hybrid and complex‐type glycans. There is extensive heterogeneity in glycosylation for each CoV, ranging from a high level of oligomannose‐type glycans (in MERS‐CoV) to highly processed complex‐type glycosylation (in SARS‐CoV) (Figure 1). Oligomannose‐type series (Man9GlcNAc2 to Man5GlcNAc2) were found concentrated in MERS S1‐NTD (spanning residues 66‐410), while SARS S1 glycans represented mostly hybrid and complex glycans (spanning residues 29‐1176).^17^, ^23^

#### 2.1.2 Host‐specific glycosylation in viral infection

Glycans function as early attachment receptors to simply tether a virus to the target cell membrane that is followed by the recruitment of secondary (co‐)receptors and endocytosis factors, as well as, the delivery of the viral genome into the cytoplasm.^4^

Host‐specific glycosylation across different individuals such as the ABO blood group system can influence viral entry and spread among individuals. For example, the presence of antibodies against the glycan structures of foreign blood can limit viral transmission from the blood group B to the blood group A individual, a selective pressure shaping the presence and distribution of viral spread. These effects have been documented in the infectivity of HIV‐1 and other viruses.^6^, ^8^

HCoV‐NL63, SARS, SARS‐CoV‐2 and MERS employ host‐functional receptors ACE2 and DPP4, respectively, for host entry and infection, but it is largely clear that functional‐receptor interactions are insufficient to allow HCoV binding and entry into the cells. Binding to surface glycans such as sialoglycans and heparan sulphates provides a measurable effect on virus adhesion and is required for viral attachment.^33^, ^34^, ^35^, ^36^

Tissue‐specific glycosylation is a key determinant of interspecies viral transmission potential and can lead to the targeting of vulnerable tissues within a host. For example, the capacity of the avian influenza virus (AIV) HA to interact with both α2,3‐ and α2,6‐linked Sia has been shown to facilitate cross‐species barrier of the virus from birds to humans. Besides, the differential expression of these Sia structures between the upper and lower respiratory tracts in humans can shape the distribution of influenza infection within an individual.^6^, ^13^, ^28^ Sialic acid‐widespread distribution of the human respiratory tract predisposes as a potential receptor and binding site for human and zoonotic viruses, as well as, their transmission.^37^ There is enough evidence that shows sialoglycans play a critical role in human zoonotic virus biology, and broaden the therapeutic options to block the replication of viruses attacking the respiratory system such as pandemic COVID‐19 and AIV epidemics.^1^, ^7^, ^10^, ^13^ Remarkably, shifting in the host of swine IVs during their adaptation is mediated by shifts in the HA Sia‐binding affinity, just as an early adaptation step of avian H9N2 strains. Only two mutations in the H9N2 AIV HA (at positions A190V and T212I), led to its adaptation to the respiratory epithelium of pigs and enhances Sia‐binding activity and virulence.^29^, ^38^ Or, a single point mutation in the highly pathogenic H5N1 AIV HA was accounted for a switch from avian enteric tract receptors (α2,3‐linked Sia) to human respiratory tract receptors (α2,6‐linked sialic acid). Modulation of a sugar‐binding site can, therefore, have profound effects on zoonotic transmission, tropism and virulence of many viruses.^2^, ^24^

Among the enveloped viruses that recognize Sia‐containing receptors are members of the families Coronaviridae, Paramyxoviridae and Orthomyxoviridae.^4^ CoV GPSs share structural features in their sugar‐binding sites (S1‐NTD) resembling the galectin‐3 folding structure in humans.^24^ A few residue changes at the sugar‐binding site can lead to efficient cross‐species infection and human‐to‐human transmission of CoVs as seen in SARS‐CoV and COVID‐19 agent.^38^, ^39^, ^40^

CoV infection requires both host and viral sialoglycans for viral attachment; HKU1 (9‐*O*‐Ac‐Sia), MERS (α2,3/6‐linked Sia, sulphated sialyl‐Lewis X), NL63 and COVID‐19 (sialylated N‐linked glycans of ACE2/S1).^6^, ^23^, ^24^, ^35^ SARS, MERS and SARS‐CoV‐2 prefer α2,3‐linked Sia, to a lesser extent α2,6‐linked Sia and sulphated sialyl‐Lewis X is the preferred binder. MERS‐CoV S1A does discriminately bind to all α2,3‐linked sialoglycans containing mono, long, branched, di‐ and triantennary Sia, with a minimum extension of 3 N‐acetyl‐D‐lactosamine tandem repeats. Specifically, the nasal epithelium of dromedary camels and type II pneumocytes in human lungs, and the intestinal epithelial cells of common pipistrelle bats widely express these kinds of sialoglycans.^1^, ^2^, ^25^ Sia distribution correlates with the upper respiratory tract infection of dromedary camels and the predominant infection of the human lower respiratory tract.^35^ Although DPP4 is expressed in the nasal epithelium of camelids, pigs and rabbits, MERS‐CoV cannot shed the respiratory tract of pigs and rabbits, unlike dromedary camels. This difference indicates that host sugars could cause interspecies variation in MERS‐CoV infection.^25^, ^35^

**FIGURE 2:**
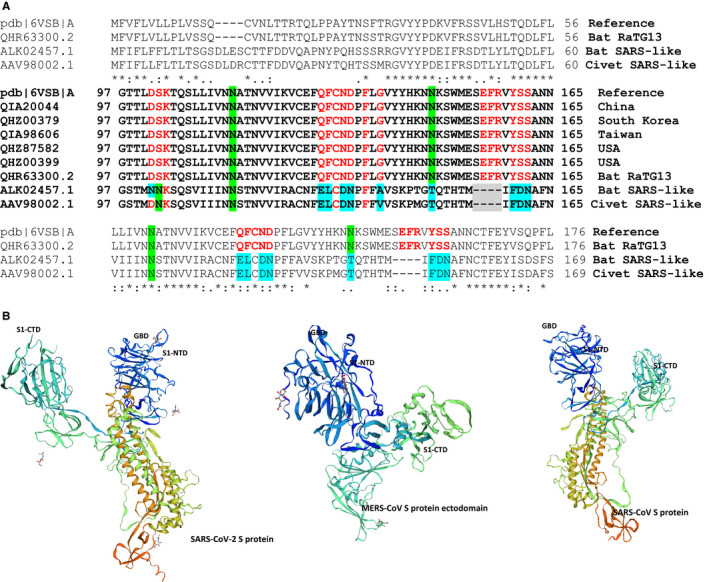
A, Amino acid sequence alignments of the ganglioside‐binding domain (GBD) of the SARS‐CoV‐2 spike protein (the reference sequence 6VSB‐A, fragment 97‐165) with clinical SARS‐CoV‐2 and Bat SARS‐like isolates (Deletions are highlighted in grey, amino acid changes in residues involved in ganglioside binding are highlighted in blue, conserved residues of GBD are lighted in red, and asparagine residues acting as glycosylation sites are highlighted in green) Structural and molecular modelling showed that amino acid residues 111‐162 of the N‐terminal domain (NTD) form a functional GBD, the interaction of which with lipid rafts can be efficiently prevented by chloroquine and hydroxychloroquine. B, Structural features of the SARS‐CoV‐2, MERS‐CoV and SARS‐CoV spike (S) proteins where the NTD could belong to a potential ganglioside‐binding domain. S1‐CTD: C‐ terminus of S1domain

The key saccharide‐binding residues are located in the S1‐NTD subdomain A which determines the host tropism and viral attachment at the early phase of viral infection.^2^, ^3^, ^24^ These residues should be strictly conserved in the Gal3‐like fold of domain A (eg Tyr162, Glu182, Trp184 and His185 in BCoV) that involved in interacting with sialoglycans in a similar way.^26^ The sialyl‐lactose binding domain of the COVID‐19 agent is identified at residues 111‐158, which is fully conserved among clinical isolates worldwide, and shows attachment to membrane lipid rafts, and facilitates contact with the ACE‐2 receptor (Figure 2).^3^, ^41^ The increased transmissibility of the COVID‐19 agent in comparison with MERS and SARS‐CoV is addressed to its engagements with sialoglycans in lipid rafts; glycoproteins and gangliosides.^3^, ^8^

##### Human sialoglycan distribution and viral infection

The N‐glycome of the human lung contains extremely large complex‐type N‐glycans with linear poly‐N‐acetyllactosamine (PL)n extensions, which are predominantly terminated in α2,3‐linked Sia. There are smaller N‐glycans lack PL while are enriched in α2,6‐linked Sia. There are also large glycosphingolipid‐glycans, which also consist of linear PL, terminating in mainly α2,3‐linked Sia.^42^ The type and distribution of Sia, and their connection to the remaining glycan structure, are highly specific for different tissues and host species. Examples of differences between tissues in the same host can be found in human airways and eyes, where Sia is usually linked to other sugars via α2,6‐glycosidic and α2,3‐glycosidic bonds, respectively. Or, there are differences in Sia distribution between the human upper and lower respiratory tract, where mainly expresses α2,6‐linked and α2,3‐linked Sia, respectively.^4^, ^37^, ^43^ There are differences in the Sia biology between humans and apes, where the expression of Sia α2,3/6‐glycosidic bonds on the airway epithelium is human‐specific and absent in the apes. Indeed, this can explain why the chimpanzee appears relatively resistant to experimental infection with human IV recognizing Sia α2,3/6‐linkages. Furthermore, while human and great ape leucocytes both express α2,6‐linked Sia, only human erythrocytes have widely expressed Sia α2,3/6‐linkages. These cell type‐specific patterns of Sia α2,3/6‐linkage expression represent an example of the human‐specific evolution of sialoglycans.^44^

Glycans containing Sia function as receptors for a large number of diverse viruses. While GlcNAc and GalNAc contain components of the scaffold, fucose and Sia are added at the end as head groups to a glycan. Sia in glycans are linked via α‐2,3 and α‐2,6 glycosidic bonds to the Gal/GlcNAc scaffold, respectively, or via α‐2,8 or α‐2,9 glycosidic bonds to other Sia.^4^, ^6^ Both α2,6‐ and α2,3‐linked Sia are widely expressed on the apical surface of human ciliated epithelium, goblet cells and submucous glands in the bronchus as well as pneumocytes of the alveoli.^37^ The α2,6‐linked Sia is mainly present in the upper respiratory tract, while the α2,3‐linked Sia is widely expressed in the lower respiratory tract. The α2,3‐linked Sia is highly expressed in the respiratory tract of young children, while the adult respiratory tract expresses mainly α2,6‐linked Sia. In comparison with adults, the neonatal pneumocytes and bronchus of children show a lower level of α2,6‐linked Sia. The human IAV strains preferentially attach to α2,6‐linked Sia, while AIV strains preferentially bind α2,3‐linked Sia. The expression profile of α2,3/6‐linked Sia on cell surfaces increases during development and maturation.^37^, ^43^ This may, in part explain why children appear to be more susceptible to AIV H5N1 than adults in the IV outbreaks. Furthermore, the sialylation increases if cells are exposed to inflammation and tumour necrosis factor. This is a reason that MERS‐CoV and SARS‐CoV‐2 cause respiratory infection in humans ranging from asymptomatic to severe pneumonia. Another reason for inter‐individual variations is due to the viral sialyl‐lactose‐containing glycoconjugates derived from the first host, and the cellular lectins, as critical determinants, located on the second host phagocytes or T cells.^6^, ^28^, ^31^, ^32^

##### Human sialoglycans and CoV trans‐infection

In immunity conditions or oxidative stress, the expression of sialoglycans, as well as, Siglec expression (Sia‐binding immunoglobulin type lectins) is significantly increased in alveolar cells which functionally associated with latter immunity responses. Increased sialylation at the terminal of glycoconjugates in the lung epithelial and monocyte cells can be a marker of chronic stress and cellular response to a stimulator whereby involving activation of recognizing receptors, changes in immune status, oxidative stress and cell turnover.^31^, ^32^, ^45^, ^46^ Structural and molecular modelling has exhibited that the amino acid residues 111‐162 of SARS‐CoV‐2 S form a functional ganglioside‐binding domain (GBD) at the tip of S1‐NTD (Figure 2), the interaction of which with lipid rafts has been reported to be prevented by chloroquine and hydroxychloroquine. The amino acid sequence alignments of the GBD of the SARS‐CoV‐2 spike protein 6VSB‐A (as the reference sequence, fragment 97‐165) with clinical isolates of SARS‐CoV‐2 show fully conserved residues among clinical isolates worldwide (Figure 2A).^3^, ^23^, ^26^

**FIGURE 3:**
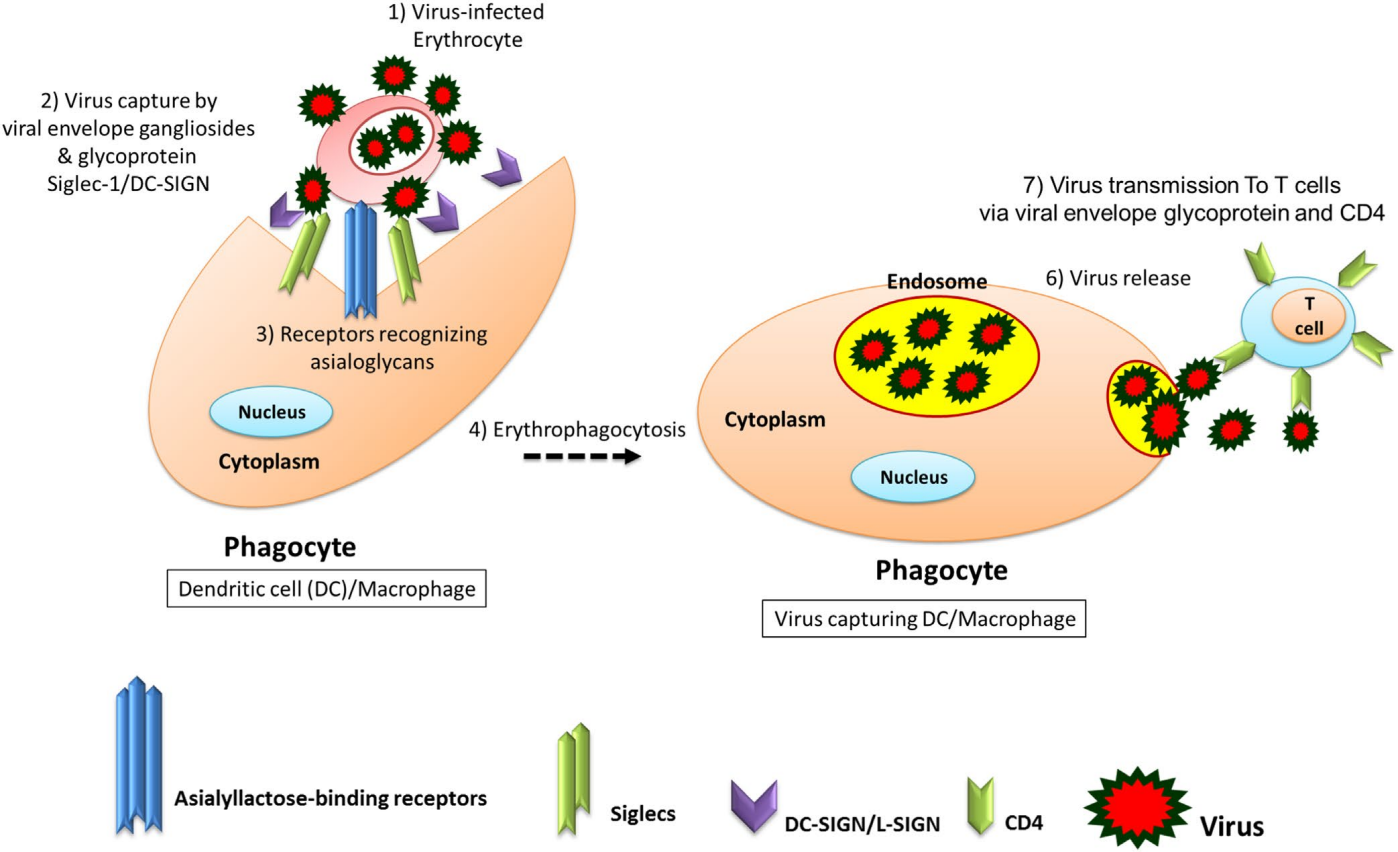
Engagement of innate immune receptors causes phagocytosis and engulfment of the virus which facilitates and/or augments infection, independently of peptidase receptors. Virus engulfment by phagocyte cells and innate immune infection through C‐type lectins. A, Virus binding and infecting an erythrocyte through gangliosides/sialogylcans on the erythrocyte, 1) Erythrocyte infection leads to changes in the glycoprofile and influences 2 & 3) phagocytosis of the infected cells, through 3) the asialoglycoprotein receptors (sialic acid‐specific lectins) located on the spleen and liver phagocytes. 2) Virus lipid membrane exposes sialyl lactose moieties for Dendritic cell (DC)/Macrophage receptors (DC‐SIGN/Siglec‐1 via recognition of the viral envelope glycoprotein and viral membrane gangliosides). 4) Viral capture is followed by 5) accumulation in a storage compartment until 6) virus is released 7) to infect a contacting CD4+ T cell via viral envelope glycoprotein and CD4/co‐receptor interactions. Immune activating signals can induce Siglec‐1 expression and contribute to virus trans‐infection. DC, Dendritic cell; ICAM‐3, specific intercellular adhesion molecule 3; DC‐SIGN, grabbing non‐integrin; Siglecs, sialic acid‐binding immunoglobulin‐like lectins

Increased expression of Siglecs in human alveolar monocytes is accompanied by an enhanced intracellular level of IL‐1β and IL‐10. Siglec‐1 interacts specifically with sialylated viral envelope proteins and gangliosides, but not with host membrane gangliosides. This mechanism likely leads to an increased viral capture and thus prolonged exposure to the cell surface receptors CD4 and CCR5 on the macrophage surface (Figure 3).^28^, ^31^, ^32^

Erythrocyte membranes are constituted with lipids, some of which are also known to promote viral fusion in T cells and virus enrichment. These lipids include sphingolipids and their glycosylated derivatives that form part of the blood group milieu of the red blood cell. In reports, lipid rafts comprising of glycosphingolipids and cholesterol were considered to be sufficient for viral fusion without the need for co‐receptors. A Sia‐binding virus that manages to make its way into the bloodstream would immediately encounter this extensive cell surface and can bind to RBCs wherein getting appropriate mechanisms to allow latter invasion and replication.^47^, ^48^ Erythrocytes are enriched in α2,6/3‐Sia residues whereby can be covered by viruses such as SARS, HIV‐1 or Zika virus and leads to erythrophagocytosis and the clearance of sialoglycan‐masked RBCs and virus capturing by liver and spleen phagocytes. Erythrocyte infection leads to reduced sialylation of GPs who are recognized by asialoglycoprotein and beta‐galactose‐binding receptors in hepatocytes and macrophages.^31^, ^47^ Herein, a preferable decoy for viruses such as SARS, SARS‐CoV‐2, IAV, HIV‐1 and Zika virus is non‐nuclear erythrocytes, widely expressing sialoglycans, representing about 50% of the total volume of blood, hiding and acting as ‘viral traps’ (Figure 3).^43^, ^44^, ^48^ Erythrocyte lysates from HIV‐1‐infected individuals have illustrated containing HIV‐1 RNA, whose plasma viral load was undetectable. The erythrocyte‐associated HIV in some of the patients exceeded that associated with leucocytes and was associated with advanced clinical stages of the disease.^48^ The sialylation pattern of glycophorin A‐C plays an important role in the invasion of RBC by various pathogens such as HIV‐1 and Zika virus and malaria parasites (ie P. falciparum), whereby the virus enrich RBCs and hides inside. Consequently, a decrease of surface sialylation (a/desialylation) occurs on RBCs which leads to their clearance and uptake by the spleen and liver phagocytes (Figure 3).^6^, ^31^, ^32^ Viruses such as HIV‐1, Zika virus and IAV, as well as, COVID‐19 agents can attack RBCs through sialoglycans expressed in the lipid rafts.^9^, ^22^

**FIGURE 4:**
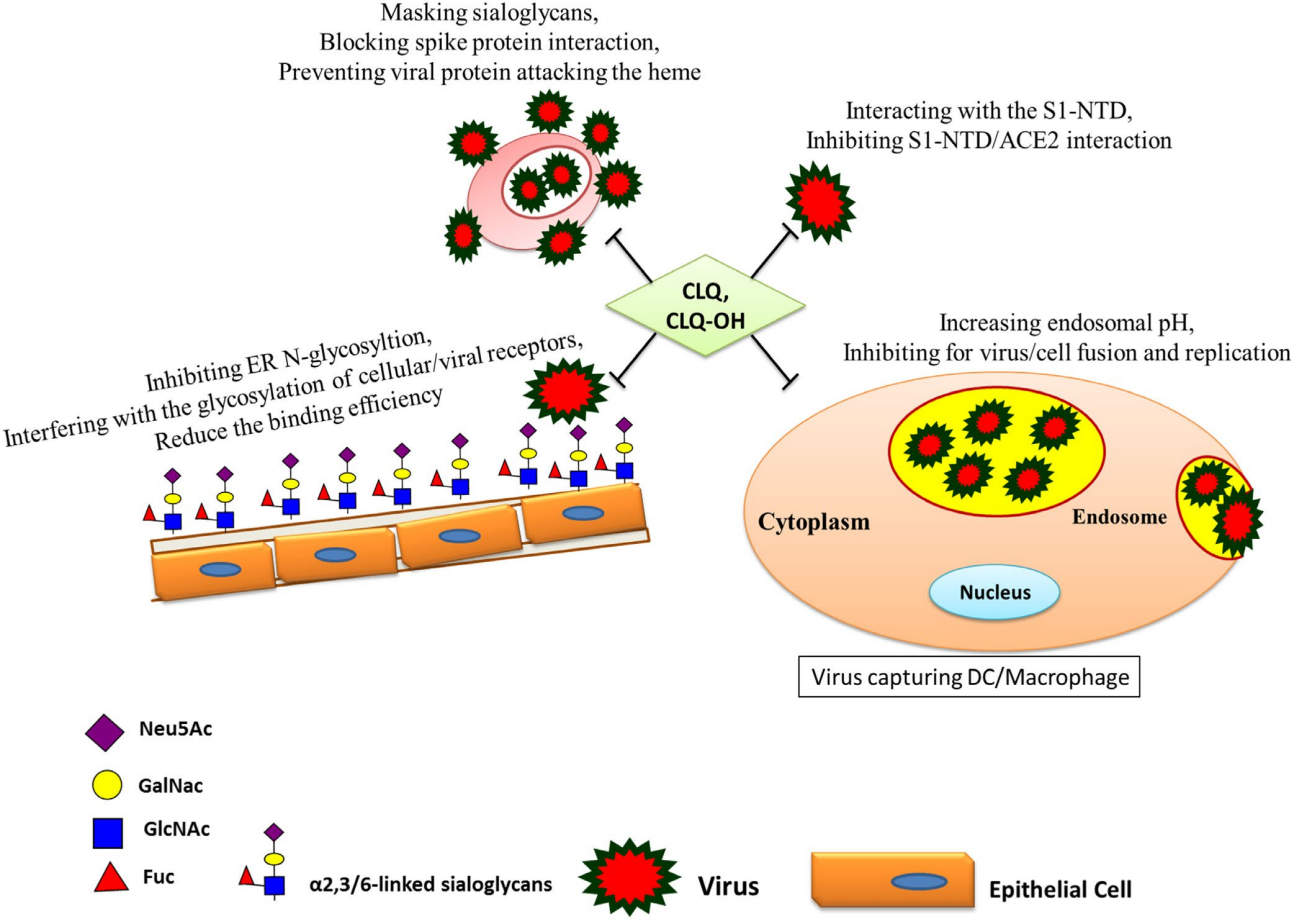
Molecular and cellular interactions of chloroquine (CLQ) and hydroxychloroquine (CLQ‐OH) with different targets that inhibit virus entrance and expansion. Dual recognition of gangliosides and sialoglycans by CoV spike (S) protein is inhibited, by masking the sialoglycans and by interfering with receptor glycosylation, the interaction between cellular lipid rafts and viral envelop is efficiently prevented

Importantly, the ganglioside‐binding domain in SARS‐CoV‐2 S1‐NTD could selectively interact with the lipid rafts of human erythrocytes and enrich infectious CoV virions in the body. The tip of S1‐NTD contains ~290 amino acids, particularly targets ganglioside‐rich microdomains in cell membranes, such as lipid rafts in RBCs. There is an over‐representation of aromatic and basic residues in the 100‐175 region of S1‐NTD including segment; 129‐ K VCE F Q F CNDP F LGV YY H K NN K S W MESE FR ‐158, which also contains Gly, Pro, and/or Ser residues. CLQ and CLQ‐OH drugs by binding to ganglioside‐binding motifs can efficiently prevent viral S1‐NTD interaction with lipid rafts of the host cell (Figure 4).^3^, ^32^

Virus binding to erythrocytes leads to erythrophagocytosis (via asialyllactose‐binding receptors or DC‐SIGN or L‐SIGN) (Figure 3). As a result, SARS‐CoV‐2 infection causes reduced levels of RBCs and haemoglobin in the blood, while leads to increased plasma concentration of free iron and hyperferritinemia.^9^, ^22^, ^31^, ^32^ Macrophage activation causes the uptake and degradation of erythrocytes (erythrophagocytosis), which leads to an iron‐overloaded capacity. The sequestration of iron within the secondary lysosomal apparatus of macrophages due to erythrophagocytosis leads to the development of a capacity for iron exocytosis. There is increased production and exocytosis of free iron of released haemoglobin, reduced tissue clearance, an increased presence of interleukin (IL)‐1β, C‐reactive protein, IL‐6, tumour necrosis factor (TNF) and interferon (IFN)‐γ and hyperferritinemia.^49^, ^50^ Accordingly, the viral attack to RBCs and temporary erythrophagocytosis cause less and less haemoglobin that can carry oxygen and carbon dioxide, as well as, more and more free iron exocytosis from overloaded phagocytes. The lung cells have extremely intense poisoning and inflammatory by the free irons and due to the inability to exchange carbon dioxide and oxygen frequently, which eventually results in ground‐glass‐like lung images.^3^, ^7^, ^22^, ^41^ Notably, the engulfment of peripheral blood cells due to the Sia masking causes the enhanced secretion of IL‐6 and TNF‐α.^6^, ^31^, ^32^ The situation may be followed by a hyper‐inflammatory life‐threatening condition in adults, associated typically with high levels of serum ferritin, evolving to multiple organ failure and unfavourable outcomes. This condition is possibly associated with adult age and with an increased presence of co‐morbidities, despite aggressive therapeutic strategies.^49^ On the other hand, viruses can exploit innate immune receptors such as dendritic cell‐specific ICAM‐3‐grabbing non‐integrin (DC‐SIGN) to infect macrophages or phagocytes whereby facilitating and/or augmenting infection. The receptors on hepatocytes or spleen macrophages recognize and bind to GPs exposing viral oligomannose and mannose or reduced levels of surface sialoglycans. This includes a wide range of membrane‐associated lectins such as DC‐SIGN, hepatic asialoglycoprotein receptor, liver/lymph node sinusoidal lectin, siglecs (binds sialylated gangliosides in viruses) and macrophage Gal/GalNAc‐specific lectin.^6^, ^28^, ^32^, ^47^ The interference of DC‐SIGN‐directed capture and transmission of HIV by CBAs has been reported.^13^ SARS‐CoV and SARS‐CoV‐2 exploit DC‐SIGN and L‐SIGN as a receptor, independently of ACE2. Seven N(Asn)‐linked glycosylation sites in SARS GPS, residues at positions 109, 118, 119, 158, 227, 589 and 699, are crucial for DC‐SIGN‐or L‐SIGN‐mediated attachment and virus entry. These residues differ from those of the ACE2‐binding domain located at amino acids 318‐510.^13^, ^28^, ^51^

It has been also confirmed by other reports that the specific glycans of the spike are recognized by the C‐type lectin receptors DC‐SIGN and L‐SIGN, and Macrophage Galactose‐type lectin (MGL)) of antigen‐presenting cells, widely present in air mucosa and lung tissue, wherein contributing to infection spread in the body.^51^, ^52^ DC/L‐SIGN, among the immune receptors, promotes virus transfer to permissive ACE2+ cells. An NMR‐based methodological approach could confirm that the RBD of the SARS‐CoV‐2 spike contains the N‐linked glycan epitopes efficiently bind to the human‐innate immune receptors Siglec, DC‐SIGN and MGL. From the data, it can be, thereby, implied that the pathogen N‐linked glycans located in the RBD of the spike protein, contain epitopes participating in the virus attachment to the innate immune receptors and its engulfment by the immune cells where can contribute to virus spread through the body.^6^, ^51^, ^52^

Herein, the virus exploits RBCs and innate immune receptors on spleen and liver phagocytes to facilitate its engulfment and capture whereby trans‐infection and syncytia formation of other cells such as T cells occurred and virus spread invasively through the body, independently of its routine functional receptor. This mechanism has been reported for viruses such as SARS, SARS‐CoV‐2, HIV‐1 and Zika virus whereby exploiting RBCs and a wide range of innate immune receptors to shifting immune responses and enriching viral infectivity.^13^, ^31^, ^32^ It is important to distinguish and differentiate the galactose‐binding receptors involved in such phenomena from soluble galectins, which can bind terminal or sub‐terminal galactose residues on cell surfaces. Galectins may function in the opposite direction, acting to reduce endocytosis of the cell surface proteins by forming lattices, and may thus be more important in the regulation of signalling, as shown by others.^6^, ^47^

## 3 TARGETING GLYCANS IN THERAPEUTICS DEVELOPMENT IN VIRAL BIOLOGY

Accordingly, the glycom of the viral envelope and the host cellular surface has a crucial role in enabling efficient transmission of the pathogen and/or entry into its susceptible target cells.^6^, ^28^

Antiviral agents that interact with the surface glycans may, therefore, compromise the efficient entry of the virus into its susceptible target cells. Such agents do not interfere with the glycosylation enzymes from the cell, but rather act by directly binding to the intact glycans on the viral and host surface. The emergence of this novel mechanism for the first step of the viral replication cycle [ie attachment to the surface of respiratory cells, mediated by the viral spike GPS] offers several potential therapeutic targets.

Carbohydrate‐binding agents (CBAs) may become the first chemotherapeutics with a dual mechanism of antiviral action: by binding the viral envelope or to the glycans of host susceptible cells subsequently blocking virus entry.^3^, ^13^, ^41^ Arbitrarily, glycan‐recognition agents (GRAs) can be categorized into two different categories of distinguished compounds: lectins, which are proteins that specifically recognize carbohydrate (glycan) structures, and non‐peptidic small‐size agents that may have a good and often specific affinity for monosaccharide and/or oligosaccharide structures.^6^, ^12^, ^13^ A category of CBAs is galectins, a group of lectins, non‐glycosylated extra‐cellular soluble proteins that specifically recognize carbohydrate beta‐galacto/sialo(glycan) structures, and their imperative and auxiliary roles have been distinguished during viral pathogenesis.^3^, ^23^, ^28^

### 3.1 Small molecules to inhibit virion attachment to surface glycans

Cell surface heparan sulphate proteoglycans (HSPGs) or other glycans provide the binding sites for CoV invasion (eg HCoV‐NL63 and SARS‐CoV), at the early attachment phase for cell entry. Lactoferrin (LF) or other glycan‐binding factors can participate to protect host defence against CoV infection through binding to glycans and blocking the preliminary interaction between CoV and host cells.^3^, ^33^, ^34^

There are suggestions for small molecules that inhibit virion attachment to HSPGs/ sialoglycans on the epithelium of the respiratory tract. For example, epigallocatechin gallate (EGCG), green tea catechin, was reported to be active against many unrelated HSPG and/or sialoglycan‐binding viruses. EGCG directly interacts with virion GPs on the surface of the virus to inhibit the attachment of herpes simplex virus type 1 (HSV‐1), hepatitis C virus (HCV), influenza A virus (IAV), vaccinia virus, adenovirus, reovirus and vesicular stomatitis virus (VSV). Additionally, EGCG competes with HSPGs for binding of HSV‐1 and HCV and with Sia for binding of IAV. Therefore, EGCG inhibits unrelated viruses by a common mechanism.^30^ Drugs CLQ and its more active derivative, CLQ‐OH, were currently reported to be effective against SARS‐CoV‐2 infection,^9^, ^41^ via binding/masking Sia on glycoconjugates and gangliosides with high affinity, as well as, via interfering with the glycosylation process in the ER and Golgi. In the presence of CLQ or CLQ‐OH, viral GPS is no longer able to bind to glycoconjugates and gangliosides (Figure 4). CLQ is a widely used antimalarial and autoimmune disease drug that has recently been reported as a potential broad‐spectrum antiviral drug (Figure 4). The identification of this new mechanism of action of CLQ and CLQ‐OH proposes the use of these repositioned drugs to prevent viral infection and cure early phase patients with SARS‐CoV‐2 infection.^3^, ^41^ CLQ by interfering with the cellular glycosylation process blocks virus entry by disrupting both viral and cellular glycosylation receptors. CLQ has an immune‐modulating activity, which may synergistically enhance its antiviral effect in vivo. Notable, CLQ is widely distributed in the whole body, including the lung, after oral administration.^7^, ^8^ Rational combination of low‐micromolar concentrations of CLQ with remdesivir effectively inhibited SARS‐CoV‐2 expansion in vitro and then in infected patients.^8^, ^9^, ^41^ Importantly, CLQ and CLQ‐OH inhibit the functional interaction between the ganglioside‐binding domain of CoV S1‐NTD with the lipid rafts of erythrocytes wherein efficiently preventing virus enrichment in the T cells and phagocytes and its body expansion.^3^, ^28^, ^32^ Additionally, CLQ could mask Sia and prevent a viral attack on RBCs and erythrophagocytosis. CLQ is described to inhibit the CoV GPS binding to porphyrins, whereby effectively relieves the symptoms of respiratory distress.^3^, ^7^, ^22^, ^41^

Importantly, the time of addition of antiviral drugs is very important wherein remdesivir was recommended to be administered at the stage of post‐virus entry, which is in agreement with its putative antiviral mechanism as a nucleotide analog. Time‐of‐addition assays for CLQ demonstrated that the drug functions at both entry and post‐entry stages of SARS‐CoV‐2 infection.^7^, ^8^

### 3.2 Galectins non‐glycosylated extra‐cellular soluble proteins, as glycan‐recognition agents

Galectins (Gal), S‐type non‐glycosylated lectins, are a subfamily of soluble/membrane‐bound proteins that typically bind β‐galactoside‐containing oligosaccharides with high specificity. Their preferred ligands should contain N‐acetyl‐lactosamine (LacNAc; Galβ1,4GlcNAc, the human blood group A‐tetrasaccharide) and related disaccharides.^53^ Gals are present in many forms of life, from nematodes and fungi to animals, where they perform a wide range of functions. Particularly in humans, different types of galectins have been described differing not only in their tissue expression but also in their cellular location, oligomerization, fold architecture and carbohydrate‐binding affinity.^21^ Viral infection induces galectin expression which participating at various levels of antiviral defence, from the initial recognition and blocking envelope and fusion glycoproteins, to the activation and amplification of the innate and adaptive immune responses.^12^, ^54^ Galectins were discovered to bind glycans on the surface of viruses, bacteria, protists and fungi.^53^, ^54^ Unlike C‐type lectins, glycan‐binding by the Gal is independent of calcium binding. Optimal use of galectins in drug production is the fact that glycosylations are not necessary for their correct folding or function, which makes it possible to scale up the production of galectins in expression systems such as *E coli*, while human‐compatible glycosylation is critical for other therapeutic GPs, as glycans can influence their yield, immunogenicity and efficacy.^6^, ^53^ Based on structural features, mammalian galectins have been classified as proto, chimera and tandem repeat types. Prototype galectins contain one carbohydrate‐recognition domain (CRD) per subunit and are usually homodimers of non‐covalently linked subunits (Gal‐1, 2, 5, 7, 10, 11, 13, 14 and 15). Chimera‐type galectins include Gal‐3 which containing a carboxy‐terminal CRD that is joined to an N‐terminal peptide and is rich in Gly‐Tyr‐Pro (Gal‐3). In the tandem repeat galectins, two CRDs are joined by a functional linker peptide (Gal‐4, 6, 8, 9 and 12).^54^ Gal‐3, in particular, can form multivalent species in a concentration‐dependent manner, in binding to β‐galactoside‐containing glycolipids and GPs exposed on the cell surface whereby leads to the formation of lattices. The 3D structure formed on the cell surface prevents endocytosis but induces signal transduction required for optimal transmission of signals relevant to cell function.^47^, ^54^

Zoonotic viruses such as IV and CoV use the Gal‐like domain to recognize and attach to the host cell surface sugars as receptors. The galectin‐like domain has been found in several viral spikes, IV HA1, rotavirus VP4, adenovirus GD and the CoV GPS S. These viral galectin‐like domains display diverse sugar‐binding modes, but their sugar‐binding sites are all located in cavities in viral RBDs, possibly to evade host immune surveillance.^14^, ^55^ Infections were identified to be initiated by the Gal‐like domain binding to a surface‐exposed sugar ligand with fast kinetics subsequently leads to GP‐mediated viral entry into the host cells.^24^, ^40^, ^56^

#### 3.2.1 Galectin‐1 (Gal‐1) and antiviral function

Gal‐1 is abundantly expressed in several kinds of cells or tissues and can be both outside and inside of cells. Gal‐1 can inhibit virus attachment and host cell fusion by binding N‐linked oligosaccharides from the virion envelope or capsid glycoproteins and promoting their cross‐linking and oligomerization and blocking cell‐cell fusion.^12^, ^54^ Numerous literature has reported the regulatory function of Gal‐1 in human virus infection, such as in HSV, HIV‐1, EBV, IV, Dengue virus, Nipah virus (NiV) and Enterovirus. Gal‐1 is an immune effector that interacts with the specific N‐glycans on the envelope fusion glycoprotein of NiV (NiVeF) and inhibits NiV infection. The binding of Gal‐1 inhibits the syncytial formation and reduces endothelial cell fusion, therefore, reducing the pathophysiologic sequela of NiV infection. Gal‐1 has shown anti‐influenza virus activity by binding to viral HA glycans and inhibiting its infectivity. An in vivo administration of Gal‐1 in mice showed that Gal‐1 lower viral load after H1N1 IAV infection. Gal‐1 can also bind to different subtypes of IAV with micromolar dissociation constant values and protect cells against IV infection and IV‐induced cell death. It is up‐regulated in the lungs of mice during IV infection and could directly bind to the envelope GPs of IAV and inhibit HA Sia‐binding and virus infectivity. More importantly, the intranasal administration of Gal‐1 could enhance the survival of mice against lethal challenge with IV by reducing viral load, inflammation and apoptosis in the lung.^12^ However, HIV‐1 can subvert Gal‐1 roles in host protection by using Gal‐1 as a vector to attach to or gain entry into host cells. Gal‐1 may function as a soluble scavenger receptor and enhance the uptake of the virus by macrophages, thereby facilitating cellular transmission of HIV‐1. There is a positive correlation between Gal‐1 levels and HIV‐1 loads during the acute phase of viral infection.^12^, ^54^

Collectively, data indicate that Gal‐1 preferentially has anti‐IV activity by binding to viral HA and inhibiting its infectivity. Thus, Gal‐1 may be further explored as a novel therapeutic agent for influenza.^12^

#### 3.2.2 Galectin‐3 (Gal‐3) and antiviral function

A survey of viral lectins with known tertiary structures revealed that galectin3‐like domains are present in a variety of viral spikes, for example, IV HA and CoV GPS.^1^, ^24^, ^55^

Gal‐3 has specific structural features in its CRDs that promote strong binding to carbohydrate ligands and high affinities in recognition features to oligosaccharide branching in particular terminated in Sia and galactose moieties. Besides its constitutive expression, Gal‐3 can be induced by inflammatory mediators, such as chemokine CXCL8.^24^, ^25^, ^40^

**FIGURE 5:**
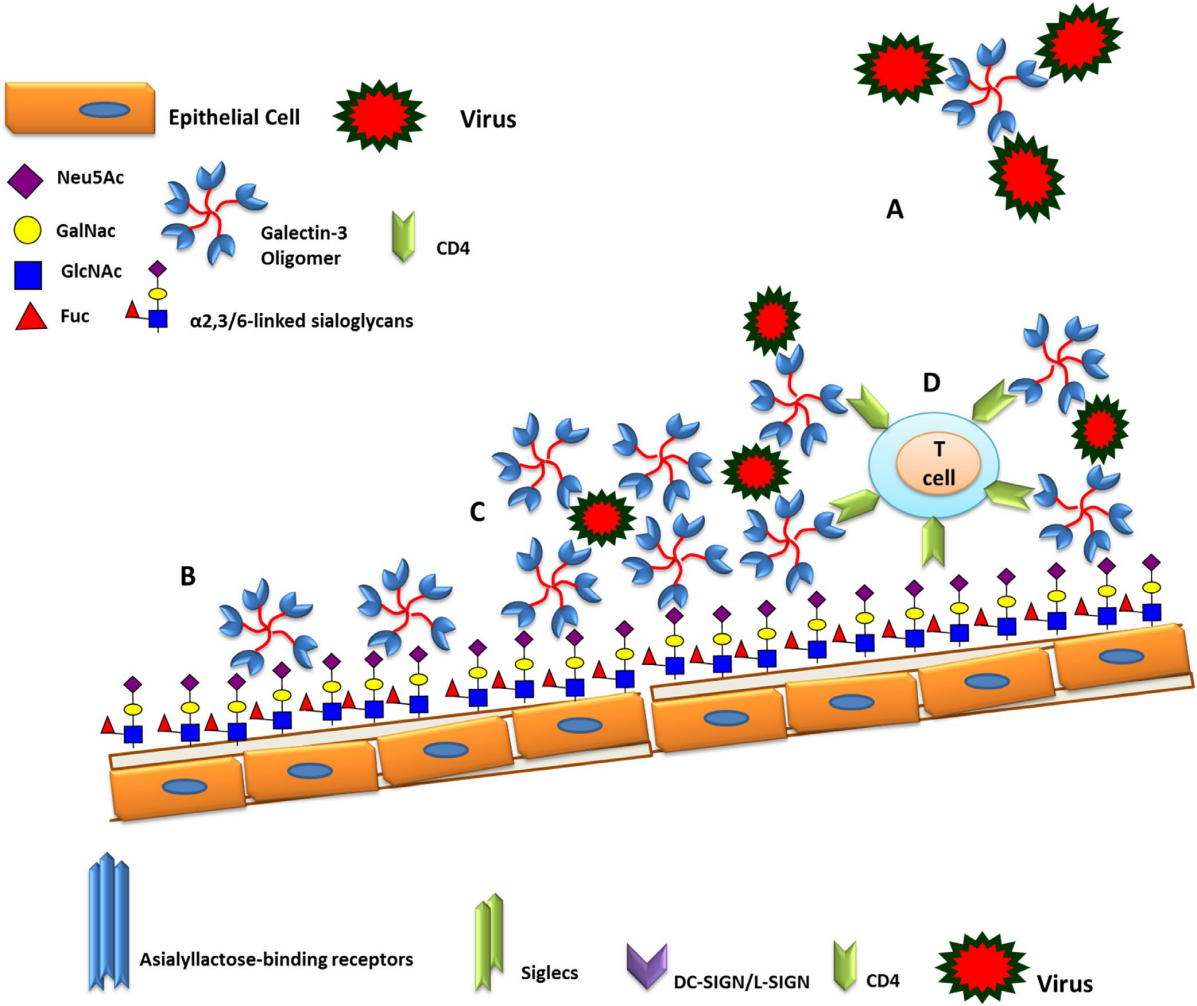
Scheme of galectin‐3 (Gal‐3) and its oligomers when interacting with glycans, as its most important cellular functions in alveolar environments and the presence of the virus. Galectin‐3 recognizes and binds terminal sialo‐lactose or sub‐terminal galactose residues (preferentially recognize α2‐3‐sialylated N‐acetyllactosamines) (A) on viral spike glycoproteins or (B) epithelial cell surfaces. Galectins function in the opposite direction, (C) acting to reduce endocytosis of the cell surface pathogens by forming lattices, and (D) being more important in regulating the immunity signalling, as shown by T cells

Gal‐3 is a 31 kD lectin, encoded by the *LGALS3* gene, expressed in the cell cytoplasm, and can be secreted onto the cell surface. In the presence of ligand and upon ligand recognition by its C‐terminal domain, Gal‐3 oligomerizes through its N‐terminal domain (forming trimers and pentamers). This oligomerization cooperatively occurs in the presence of multivalent oligosaccharides in solution or at the viral surface and leads to lattice formation and cross‐linking of GP receptors on the cell surface, which is an essential event for T cell activation and the host defence functions but not endocytosis (Figure 5).^11^, ^21^, ^47^, ^57^

During viral infection, Gal‐1 displays anti‐inflammatory features, remains a dimer in cross‐linked complexes with a bivalent oligosaccharide and inhibits leucocyte infiltration, whereas Gal‐3 displays pro‐inflammatory activity, organizes supramolecular assemblies and enhances macrophage survival and recruitment. The functional activity of a pro‐ or anti‐inflammatory galectin could be explained in terms of lattice formation and the quaternary structure, the organization of supramolecular assemblies. Due to its dimeric equilibrium, Gal‐1 can form one‐dimensional and homogeneous lattices, while Gal‐3 forms heterogeneous cross‐linked complexes with multivalent carbohydrates.^11^, ^21^ Observation highlights the relevance of the subtle differences in galectin specificity and affinity that may determine the very different pathogen recognition outcomes. Evidence shows that the chimera‐type Gal‐3 can inhibit viral adhesion to epithelial cells.^11^, ^57^ Macrophage or CD4 T cells attacked by HIV‐1, up‐regulate Gal‐3 whereby participating in antiviral immunity. Gal‐3 does not affect HIV‐1 adsorption, entry or infection.^12^, ^54^ Gal‐3 levels have been described as a biomarker of HSV and dengue virus infection correlated with macrophage polarization. Additionally, in end‐stage renal disease (ESRD) with chronic inflammation and high levels of IL‐6, Gal‐3 up‐regulation seems to protect against the ongoing pro‐inflammation and reduce chronic inflammation.^11^ Even more, Gal‐3 knockout mice display age‐dependent increased adiposity, dysregulated glucose metabolism and systemic inflammation, including accelerated diabetes‐associated kidney damage and diet‐induced atherogenesis.^57^ Like other galectins, Gal‐3 can also participate to inhibit the cell fusion and syncytial formation mediated by viral envelope GP through binding to specific N‐glycans. Syncytia formation leads to the body expansion of SARS, MERS, COVID‐19 novel agent and HIV infection. The formation of giant, multinucleated cells or syncytia has been proposed as a strategy to allow direct spreading of the virus between cells, subverting virus‐neutralizing antibodies.^12^, ^28^, ^29^, ^36^

##### Gal‐3 as therapeutic agents binds to respiratory glycans

Gal‐3, as a pattern recognition receptor (PRR), plays potential roles in the microbe's recognition by the host and promoting the host's immune responses. Gal‐3 is supposed to participate in the regulation of antiviral immunity. The mechanism has been illustrated by the participation of Gal‐3 in the infection inhibition of HIV.^53^, ^57^ Regarding airway viral infection, CoVs and IAV use the Gal‐3‐like domains in their spike GPs (GPS and HA, respectively) to bind to sialoglycans on the apical surface of epithelial cells. Gal‐3 uses both virus and host glycans to recognize and binds to Sia‐containing receptors in the airway whereby infection or entry of virus to the body is inhibited. In masking the Sialo‐galacto‐glycans, the spike GPs cannot bind the host receptors that could block virus attachment and human respiratory epithelium infection.^15^, ^24^, ^40^, ^58^ From the literature, it is interpreted that intranasal administration of Gal‐3 before or during the early phase of virus infection would reduce viral load, accompanying inflammation, tissue damage and mortality in the susceptible host.^11^, ^12^, ^59^ In a mouse model for IAV infection, Gal‐3 was discharged on the airway epithelia and caused the release of pro‐inflammatory mediators that induce the expression of antiviral genes, including IFNs (β & γ), to inhibit IAV replication.^11^ As intranasal administration of recombinant Gal‐1 in infected mice led to reducing viral load, accompanying inflammation, tissue damage and mortality in the murine model infected with influenza virus.^15^, ^58^

The protective effect of Gal‐3 at the airway epithelia against CoV and IV infection can be through promoting viral cross‐linking and oligomerization while blocking viral‐cell fusions. By binding to Sialosaccharides on the envelope GP or airway epithelia, Gal‐3 prevents syncytia formation, while promoting the organization of surface mucins to maintain mucosal barrier function in the airway.^58^, ^59^, ^60^ Gal‐3‐virus attachment induces pro‐inflammatory reactions and protective innate or adaptive immune responses against the pathogen. For example, the binding of viral glycotopes to Gal‐3 which is attached to cell surface mucins and associated with TLR2 leads to TLR2‐mediated production of tumour necrosis factor‐α (TNF‐α) and IL‐1β by the immune cells in the epithelium barrier. As TNF‐α and IL‐1β are critical cytokines that promote parasite clearance, the direct interaction of Gal‐3 and pathogen glycotope is an important initial step to reduce the extent of infection.^53^, ^54^, ^56^

## 4 CONCLUSION

Considering, however, the host‐targeted approach could have some major limitations while designing drugs against COVID‐19 in terms of clinical implementation. The paramount concern is the toxicity underlying the ‘on‐target’ suppression of host functions required for viral replication. Because inhibiting host glycosylation machinery disrupts the glycan processing of both viral and host cellular glycoproteins. For example, alteration of N‐linked glycans may disrupt ACE2 interaction with other cellular component(s) that might facilitate the membrane fusion between the virus and host cells. Or clinical implementations of CBA as host‐targeted approaches need also some concerns. There are reports for pro‐ and antiviral consequences of soluble lectin interactions or pro‐ or anti‐inflammatory roles that they play in the innate immune system. The diverse, at times, paradoxical roles that they may play in the innate immune system are related to their different interactions at the molecular level which is dependent on their structure‐activity relationship and needs to be dissected.^5^, ^6^, ^11^, ^21^

Thereby, surface glycans as an important class of microbial signature that is recognized by a variety of host cell lectins can function as both recognition and effectors against viral and bacterial infection. In therapeutic design, surface glycans can ‘subvert’ viral adhesion to or cause to gain entry into the host cells. Herein, host‐intranasal administration of a galectin, before or during the early phase of virus infection, is proposed that recognize specific glycan ligands and subverting viral adhesion and promoting host immune response. Data suggest that galectins (here in Gal‐1 and ‐3) can potentially be used as viral therapeutic targets or antagonists.

## CONFLICT OF INTEREST

There is no conflict of interest to be declared.

## AUTHOR CONTRIBUTIONS

**Fatemeh Pourrajab:** Conceptualization (equal); Data curation (equal); Formal analysis (equal); Investigation (equal); Project administration (equal); Visualization (equal); Writing‐original draft (equal); Writing‐review & editing (equal).
